# The Enigma of Endothelium in COVID-19

**DOI:** 10.3389/fphys.2020.00989

**Published:** 2020-08-04

**Authors:** Savneet Kaur, Dinesh M. Tripathi, Angeera Yadav

**Affiliations:** ^1^Department of Molecular and Cellular Medicine, Institute of Liver and Biliary Sciences, New Delhi, India; ^2^Department of Pharmacy Practice, National Institute of Pharmaceutical Education and Research, Guwahati, India

**Keywords:** COVID-19, endothelial dysfunction, pathogenesis and diagnosis, viral sepsis, inflammation, coagulation, vascular biology

## Abstract

Coronavirus disease 2019 (COVID-19), caused by severe acute respiratory syndrome–related coronavirus-2 (SARS-CoV-2) has affected millions of people globally. Clinically, it presents with mild flu-like symptoms in most cases but can cause respiratory failure in high risk population. With the aim of unearthing newer treatments, scientists all over the globe are striving hard to comprehend the underlying mechanisms of COVID-19. Several studies till date have indicated a dysregulated host immune response as the major cause of COVID-19 induced mortality. In this Perspective, we propose a key role of endothelium, particularly pulmonary endothelium in the pathogenesis of COVID-19. We draw parallels and divergences between COVID-19-induced respiratory distress and bacterial sepsis-induced lung injury and recommend the road ahead with respect to identification of endothelium-based biomarkers and plausible treatments for COVID-19.

## Introduction

Endothelium is a highly specialized and dynamic organ which serves numerous roles in both physiology and pathophysiology (Galley and Webster, 2004). Being the first organ to perceive any damage to the underlying tissue, it effectively adapts and mounts compensatory responses to any chemical, mechanical and cellular injury. The pulmonary endothelium that shields the lungs comprises of both macro or microvascular endothelial cells with considerable phenotypic and functional heterogeneity (Aird, 2007). Pulmonary microvascular endothelial cells (mEC) are an integral part of the alveolar (epithelial)-capillary (endothelial) barrier formed by tight junctions and adherens junctions in the lungs. This impermeable alveolar-capillary barrier strictly restricts vascular fluid fluxes across the lung epithelium and hence the integrity of this barrier is utmost to prevent pulmonary edema, congestion and respiratory failure (Millar et al., 2016). In the current perspective, we envisage a key role of mEC in the pathogenesis of coronavirus disease 2019 (COVID-19) caused by the novel coronavirus, severe acute respiratory syndrome–related coronavirus-2 (SARS-CoV2).

## COVID-19 and Pulmonary Inflammation

COVID-19 has a wide clinical spectrum ranging from asymptomatic to mildly symptomatic forms to severe clinical conditions such as respiratory failure, sepsis and multiorgan dysfunction syndromes (MODS) (Yang X. et al., 2020). Most of the hospitalized critically ill COVID-19 patients have pneumonia with abnormal chest CT scans and acute respiratory distress syndrome (ARDS) seems to be the major causes of death in these patients (Yang F. et al., 2020). ARDS is nothing but a reflection of severe mEC dysfunction involving changes in vascular permeability, inflammation, accumulation and extravasation of leucocytes, activation of procoagulant pathways and disruption of alveolar-capillary barrier (Matthay et al., 2019). Evidence from several studies have indicated that patients with COVID-19 exhibit most of these attributes. Lung pathology from COVID-19 patients has depicted that along with pulmonary type 2 alveolar epithelial cells, endothelial cells in the systemic venules are also desquamated and an inflammatory reaction is present in blood vessel walls (vasculitis), suggestive of intense vascular reactions (Ding et al., 2003). Lung autopsies from COVID-19 patients also show massive neutrophil infiltration in pulmonary capillaries, acute capillaritis and extravasation of neutrophils into the alveolar space (Fox et al., 2020; Liu et al., 2020). Pulmonary neutrophilia is predictive of poor clinical outcomes and also neutrophil-to-lymphocyte ratio is an independent risk factor of disease severity in these patients (Liu et al., 2020). An inflamed/injured endothelium due to increased expression of adhesion molecules, including E- and P-selectins, loss of cadherin junctions and hence altered vascular permeability promotes the adhesion and migration of neutrophils. Neutrophilia can induce injury to the endothelium-epithelial alveolar barrier, causing further damage to the lungs. A recent report has illustrated that neutrophil extracellular traps (NETs) are enhanced in hospitalized COVID-19 patients receiving mechanical ventilation as compared with hospitalized patients breathing room air. The study has also documented that sera from individuals with COVID-19 trigger NET release from control neutrophils *in vitro* (Zuo et al., 2020). NETs are extracellular DNA fibers of neutrophils carrying nuclear proteins, such as histones and bactericidal proteins. NET formation in the lungs is documented in bacterial sepsis and thrombosis, however, their role in viral infections is not very well known except for a report which has shown their presence in influenza virus-induced pneumonia *in vivo* (Narasaraju et al., 2011). NETs represent one of the powerful host mechanisms to damage the microbes. They are, however, suicidal, if present in excess as they can attach to the capillary endothelium, collate with the platelets to induce coagulation and thus cause damage to the alveolar–capillary barrier, leading to vascular leakage, edema and finally ARDS (Sørensen and Borregaard, 2016). Ascertaining the role of NETs as markers of COVID-19 disease severity needs further systematic studies and targeting of NETs to rescue the lung endothelium represents a worthwhile therapeutic option in COVID-19.

## COVID-19-Induced Atypical ARDS

Several studies have indicated the presence of atypical manifestations of ARDS in some severe patients of COVID-19. They exhibit near normal pulmonary compliance of >50 ml/cmH_2_O with severe hypoxemia, which seems to emanate due to impaired hypoxic pulmonary vasoconstriction (HPV) and ventilation/perfusion (V/Q) mismatch (Gattinoni et al., 2020). An impaired HPV in these patients point toward mEC dysfunction in the lungs as recent studies have postulated that the alveolar ion channels in the endothelial cells of the alveolar-capillary barrier are sensors of oxygen/hypoxia that propagate the messages along the vascular endothelium and initiate arteriolar vasoconstriction or HPV (Grimmer and Kuebler, 2017). These patients show features of vasoplegia or persistent hypotension as observed in sepsis patients with atypical ARDS, indicative of a primary insult to the pulmonary endothelium (Gattinoni et al., 2020). Lung histology in COVID-19 patients with severe respiratory failure also indicates that COVID-19 has features distinct from typical ARDS (Klok et al., 2020). The pulmonary abnormalities in these patients comprise of thrombotic microvascular injury in the alveolar capillaries, with fewer signs of viral cytopathic or fibroproliferative changes. Pulmonary thromboembolic complications with a distinct pro-coagulant profile, elevated D-dimer levels and angiogenesis is a common finding in many severe cases of COVID-19 patients (Ackermann et al., 2020; Ranucci et al., 2020). Increased ratio of angiogenic factor, soluble fms-like tyrosine kinase 1 (sFlt-1)/PLGF (placental growth factor) ratio in COVID-19 positive patients as compared to patients with COVID-19 negative pneumonia, and healthy donors is recently documented (Giardini et al., 2020). The use of the s-Flt1/PlGF ratio in COVID-19 as a clinical tool to stratify the intensity of endothelial dysfunction has been proposed in this study.

A healthy lung endothelium has an inhibitory effect on inflammation and coagulation while a procoagulant phenotype is a hallmark of an injured endothelium in sepsis and ARDS both (Millar et al., 2016). These studies along with the fact that the pulmonary epithelium is more resistant to injury than the endothelium signify that SARS-CoV-2-induced ARDS and associated coagulopathy may be caused by a direct endothelial infection by the virus in the lungs (Matthay et al., 2019). Another study on a few post-mortem biopsies has, however, illustrated that SARS-CoV-2 nucleocapsid protein immunopositivity is not observed in the mECs and is present only in lung pneumocytes and ciliated epithelial cells (Schaefer et al., 2020). Interestingly, in this study, five patients also exhibit blood clots in the pulmonary vasculature. In absence of a direct pulmonary endothelial involvement and viral infection, this may be explained as diffuse pulmonary intravascular coagulopathy, which is distinct from sepsis-induced disseminated intravascular coagulation (Marchandot et al., 2020; McGonagle et al., 2020). However, an absence of viral positivity in the mECs in this study can also be attributed to technical limitations. Further studies and sensitive imaging techniques are needed to delineate if SARS-CoV2 causes a direct endothelial injury as seen in sepsis-induced ARDS or a direct epithelial injury as in typical ARDS. Both these conditions have different pathological hallmarks and hence can be dissected. A direct endothelial injury can be characterized by increased levels of Angiopoetin-2, von Willebrand factor (vWF), Soluble thrombomodulin, Interleukin 8, Soluble ICAM-1 indicating massive endothelial stimulation and damage, while direct epithelial injury can be identified by high plasma levels of surfactant protein-D and receptor for advanced glycation end products (Hendrickson and Matthay, 2018).

## Entry of COVID-19 via Pulmonary Endothelial Cells

In case of direct injury to mEC, SARS-CoV-2 should enter the lungs via the mECs. Angiotensin-converting enzyme 2 (ACE2), the type I integral transmembrane protein, a functional receptor for SARS-CoV and a potential receptor for SARS-CoV-2 is highly expressed in the mEC, along with the lung epithelial cells (Hamming et al., 2004; Hoffmann et al., 2020). Although the contribution of ACE2 in the pathogenesis of COVID-19 is not known, yet it has been delineated that SARS-CoV infection downregulates ACE2 and worsens lung injury that is reversed by treatment with Angiotensin receptor blocker-mediated upregulation of ACE2 (Imai et al., 2005). This study and several others show a protective role of ACE2 in ARDS and lung injury. A recent study has also revealed that the use of human recombinant soluble ACE2 prevented host cell binding to SARS-CoV-2, probably by viral binding to proteins in solution rather than those on host cells (Monteil et al., 2020). However, the precise *in vivo* role of mEC-specific ACE2 vis-à-vis circulating ACE2 levels in COVID-19 infection and/or severity, especially in context of their vascular effects demand stringent evidence-based studies. Besides ACE2, other receptors such as CD209L and vimentin that have been proposed to serve as putative receptors of SARS viruses are also expressed in mEC (Jeffers et al., 2004; Yu et al., 2016).

Another possibility worth mentioning is that SARS-CoV-2 may also be transported to mEC via air-borne particulate matter with a diameter of 2.5 micrometers (PM2.5) or smaller. These fine air particles can easily reach the smallest of the human airways, cross the alveolar-capillary barrier, deposit on vascular endothelium via specific receptors, modulate vascular permeability and facilitate systemic inflammation, also leading to coagulation (Wang et al., 2017). Recent reports have indeed shown a positive correlation between the increased presence of air pollutants (PM2.5) and COVID-19 spread and lethality (Fattorini and Regoli, 2020). Although the air-borne spread of SARS-CoV-2 via PM and its direct entry into mEC remains to be established, it would be interesting to unravel molecular mechanisms if and how SARS-CoV-2 survive and thrive in the mEC.

## COVID-19 and Sepsis

COVID-19 infection also seems to mount an attack and exacerbate endothelial damage in other vascular beds (Sardu et al., 2020a). Active SARS-CoV-2 replication in human capillary organoids that closely resemble human capillaries has been demonstrated, suggestive of the fact that the virus could directly infect ACE2-positive blood vessel cells (Monteil et al., 2020). Presence of viral elements within the endothelial cells and diffuse endothelial inflammation has been documented in post-mortem biopsies of lung, heart, kidney, skin and liver (Colmenero et al., 2020; Varga et al., 2020). These studies suggest that a direct SARS-CoV-2 infection facilitates endothelial injury in other vascular beds besides the lungs. However, since these are post-mortem biopsies of patients with respiratory failure, it is probable that endothelial damage at this phase is because of an overwhelmed host inflammatory response, rather than viral replication and increased viral loads in the endothelial cells.

A high mortality rate and poor clinical outcomes due to COVID-19 infections in aged subjects and patients with comorbid conditions including diabetes, obesity and hypertension may also be because of an underlying endothelial dysfunction (Yang J. et al., 2020). Indeed, certain features of endothelial dysfunction such as altered permeability, imbalance between vasoconstrictors and vasodilators, markers of pro-coagulation that are well known to be present in all the above conditions, would serve as key indicators for identifying those subjects who are at a higher risk of developing severe form of the disease. Large-vessel stroke has been described in young patients of COVID-19 with a mean stroke scale of 17 indicating a large area of ischemia. All of them had to be given clot retrieval therapy, anticoagulation and antiplatelets (Oxley et al., 2020). Three patients in this study had diabetes indicating that an underlying endothelial damage might be one of the key reasons of SARS-CoV-2-induced stroke in these patients. Stroke and thrombotic events in brain could also be, however, ascribed to viral infection-induced thrombophilia. The nucleocapsid and spike proteins of the SARS-CoV-2 have been speculated to contribute to a pro-thrombotic state due to their modulation of clotting pathways in the lungs via dysregulation of ACE2 (McGonagle et al., 2020).

The organotropism of SARS-CoV-2 beyond the respiratory tract has been seen in autopsy studies. Besides lungs, viral loads and extensive inflammation have been spotted in the kidneys, liver, heart and brain implicating direct viral tissue damage (Puelles et al., 2020). Given the dissemination of the virus to other organs and presence of MODS in some patients, Li et al. (2020) has conceived the term “viral sepsis” for severe COVID-19 infections. Although systemic inflammation may also occur in the wake of an exuberant host cytokine response, given the presence of viral elements in extrapulmonary tissues, it is reasonable to hypothesize that SARS-CoV-2 infection might be spreading through the blood cells and the endothelial route may be one important factor facilitating this viral spread via blood. However, this hypothesis needs further investigations as studies have reported less or even negligible amount of viral RNAemia in infected patients (Huang et al., 2020; Wölfel et al., 2020). More advanced and sensitive diagnostic techniques and the culture of SARS-CoV-2 in blood cells would be needed to confirm the hematogenous spread of viral infection. Here, it is also to be noted that most of the severe COVID-19 patients with ARDS develop bacterial coinfections in the hospitals, thus complicating the true clinical picture (Zhou et al., 2020). It is thus pertinent to clearly distinguish patients with only SARS-CoV2 infections from those having SARS-CoV2 plus secondary bacterial infections. A distinguishing marker could be serum procalcitonin (PCT) levels. Unlike patients with bacterial sepsis, PCT values would remain within the reference range in viral sepsis including severe COVID-19 patients as PCT synthesis is known to be inhibited by interferon-gamma released during viral infections (Lippi and Plebani, 2020). A recent elegant study has identified unique immune signatures in severe COVID-19 patients, characterized by a high sustained cytokine production of interleukin-6 (IL-6), a pleiotropic cytokine with multiple effects in these patients, distinct from patients with bacterial sepsis. COVID-19 patients also show IL-6-mediated low HLA-DR expression and lymphopenia (Giamarellos-Bourboulis et al., 2020). Further such large-scale studies would shed light on how COVID-19-mediated sepsis is similar and/or different from bacterial sepsis to avoid overlapping of the two distinct diseases.

## Perspectives

**FIGURE 1:**
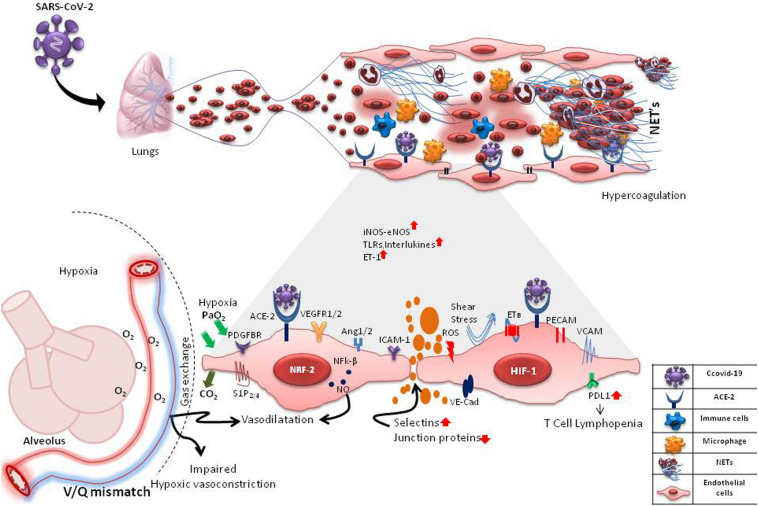
Probable Interactions of SARS-CoV-2 with pulmonary microvascular endothelial cells (mECs). SARS-CoV-2 may interact with the mECs directly via ACE2 receptor or indirectly affecting a multitude of endothelial-mediated functions including changes in intercellular permeability, expression of selections, adhesins and VE-cadherins, changes in expression of angiogenic and other functional receptors such as VEGFR1/2, PDGFBR, S1P_2/4_, PECAMs, ICAMs, VCAM1 causing neutrophilia, formation of neutrophil extracellular traps (NETs), inflammation, hypercoagulation and hypoxia. Hypoxia causes induction of hypoxia-inducible factors (HIF-1) and angiogenesis. ROS from activated neutrophils induces expression of transcription factors, Nrf2 and NFk-B, causing further inflammation. These changes in mECs result in an increase/decrease of vaso-active mediators like nitric oxide (NO), endothelin-1, oxygen sensing machinery, impaired hypoxic vasoconstriction and V/Q mismatch. In inflammatory states, the mECs are also subjected to disturbed blood flow (shear stress). mES dysfunction may also cause an increase in the expression of PD-L1, leading to T cell lymphopenia. These drastic changes in the capillary endothelium disrupts the alveolar-capillary barrier, causing edema and finally respiratory distress.

Directly or indirectly, endothelial cells and particularly mECs are a crucial link between SARS-CoV-2 and host immune responses and thus may serve many roles in determining the disease severity and mortality in COVID-19 (Figure 1). As the clinical, pathological and molecular features of COVID-19 patients are unfolding in investigational studies, several researchers are hypothesizing and reviewing a vascular-centric pathogenesis of COVID-19. A summary of such recent reviews and short reports is provided in Table 1 (Alvarado-Moreno and Majluf-Cruz, 2020; Amraei and Rahimi, 2020; Cure and Cure, 2020; Froldi and Dorigo, 2020; Guler et al., 2020; Gupta et al., 2020; Gustafson et al., 2020; Mangalmurti et al., 2020; Marchetti, 2020; Mondal et al., 2020; Panfoli, 2020; Pons et al., 2020; Sardu et al., 2020b; Teuwen et al., 2020). Hence, undoubtedly, an assessment of endothelial dysfunction in patients with COVID-19 to stratify the patients based on severity holds immense relevance. Circulating biomarkers such as endothelin-1, E- and P-selectins, vWF and soluble adhesion molecules that signify endothelial dysfunction may appear as early biomarkers of viral infection and probable organ dysfunction (Goshua et al., 2020). Pulmonary endothelium has the highest expression of the angiogenic factors like, vascular endothelial growth factor (VEGF) and a dysregulated pulmonary angiogenesis is a well-known mediator of acute lung injury (Wada et al., 2013). An evaluation of angiogenic factors and their soluble receptors, particularly sVEGFR2 and Angiopoetin-2, might be of prognostic significance in these patients. Circulating endothelial progenitor cells (EPCs) may also be used to assess/predict COVID-19 disease severity and progression in longitudinal studies as altered levels of EPCs have been demonstrated in patients with both acute lung injury and bacterial sepsis (Witzenrath, 2014). Identification of biomarkers and molecular mechanisms underlying COVID-19-induced endothelial injury should lead to new pharmacological targets to ameliorate several processes starting from vascular permeability to neutrophil accumulation, angiogenesis, pro-coagulation to V̇/Q̇ mismatching in COVID-19 pathogenesis. Meanwhile, a variety of currently used or investigational drugs such as statins, tyrosine kinase inhibitors, atrial natriuretic peptide, S1P_1_ agonists that exert endothelial protective or repair effects may be explored for their effects in COVID-19. Dexamethasone, a synthetic corticosteroid and other anti-inflammatory drugs that are already used in many inflammatory diseases including sepsis and ARDS seem to be a ray of hope in mitigating mortality in critically ill patients of COVID-19 (Ledford, 2020). Dexamethasone and other steroids are also known to inhibit endothelial activation and levels of soluble VCAM1 and E-selectins in both *in vitro* and *in vivo* models of sepsis (Zielińska et al., 2016). The precise effects of steroid treatment on endothelial function in COVID-19 patients with varying severity thus warrant in-depth investigations.

**TABLE 1:** Summary of studies hypothesizing an endothelial-specific pathogenesis of COVID-19.

**S. No**	**Major views**	**Type of study**	**Study (References)**
1.	The endothelium is a key target organ in COVID-19 and a major determinant of disease severity.	Review	(Sardu et al., 2020b)
2.	Deaths occurring in Mexico at younger age as compared with other countries may be related to the high frequency of vascular risk factors and the consequent endothelial dysfunction.	Opinion	Alvarado-Moreno and Majluf-Cruz, 2020
3.	Key role of endothelial dysfunction during SARS-CoV-2 infection, as a direct target of the virus and inflammatory cytokines.	Review	Pons et al., 2020
4.	Binding of virus to ACE2-positive endothelial cells may cause production of ROS, which cause these cells to acquire a pro-thrombotic and pro-inflammatory phenotype, predisposing patients to thromboembolic and vasculitic events and to disseminated intravascular coagulopathy.	Short Report	Panfoli, 2020
5.	Comparative study between various coronavirus reveals similarities in entry and pathogenesis of SARS-CoV-2 along with SARS and MERS in various aspects of multi organ involvement and systematic vasculitis along with similarities in immune-pathogenesis. The involvement of multiple organs in COVID-19 indicates that endothelial injury leading to visceral vasculopathy may be the inciting factor in the disease progression.	Review	Mondal et al., 2020)
6.	Vascular-centric pathology of COVID-19-induced ARDS. Complement pathway activation and ACE2 dysregulation contributing to vascular injury and thrombosis in COVID-19.	Hypothesis and Review	Mangalmurti et al., 2020)
7.	Passing of SARS-CoV-2 from the respiratory epithelium to the endothelium for viral dissemination. Cytokine-driven vascular leak in the lung alveolar-endothelial interface promotes acute lung.	Review	Gustafson et al., 2020
8.	ACE2 and RAS signaling, as a possible link between the pre-existing endothelial dysfunction and SARS-CoV-2 induced endothelial injury in COVID-19 associated mortality. Key roles of endothelial cell-expressed cell adhesion molecules including CD209L/L-SIGN and CD209/DC-SIGN in SARS-CoV-2 infection.	Review	Amraei and Rahimi, 2020
9.	Serum Angiotensin II levels are increased with ACE2 blocking by COVID-19. Angiotensin II stimulation and local stimuli such as H^+^ ion and hypoxia activate Na^+^/H^+^ exchanger (NHE) in both vascular endothelium and platelets. COVID-19 can lead to thrombosis by causing NHE activation.	Letter	Cure and Cure, 2020)
10.	Apoptotic processes in the ACE2-positive lung microvascular bed endothelium or coronary endothelium by SARS-CoV-2. Increased D-dimer may be the result of apoptotic endothelial cell induced coagulopathy.	Letter	Guler et al., 2020
11.	Endothelial damage mediated by complement activation, inflammation, hypoxia, platelets, and ABL tyrosine kinases as relevant cause of death in patients with COVID-19.	Review	Marchetti, 2020)
12.	Endothelial cells play a central role in the pathogenesis of ARDS and multi-organ failure in patients with COVID-19.	Review	Teuwen et al., 2020
13.	Increased mortality in young males in Italy linked to the diversity in sex-hormones. Activation of endothelial estrogen receptors increases nitric oxide and decreases ROS, protecting the vascular system of females from angiotensin II-mediated vasoconstriction, inflammation, and ROS production.	Hypothesis	Froldi and Dorigo, 2020)
14.	Extrapulmonary organ-specific pathophysiology and clinical presentation of COVID-19. Central role of endothelial damage and thrombo-inflammation.	Review	Gupta et al., 2020

*ROS, reactive oxygen species; ACE2, angiotensin converting enzyme2; ARDS, acute respiratory distress syndrome.*

## Conclusion

To summarize, the pulmonary endothelium seems to be standing at the crossroads of COVID-19, forming the very crux of this baffling disease. Endothelial dysfunction may be both a cause and/or effect of severe COVID-19. It is thus imperative to have further insights into this entity to embark upon newer prognostic markers and effective treatment options for COVID-19 and in fact for viral sepsis in general.

## Data Availability Statement

The original contributions presented in the study are included in the article/supplementary material, further inquiries can be directed to the corresponding author.

## Author Contributions

SK developed these perspectives. SK and DT together drafted the manuscript. AY provided support in conceiving and designing the figure under the supervision from DT. SK finalized the manuscript. All authors contributed to the article and approved the submitted version.

## Conflict of Interest

The authors declare that the research was conducted in the absence of any commercial or financial relationships that could be construed as a potential conflict of interest.
